# Retraction Note: pH-responsive chitosan/acrylamide/gold/nanocomposite supported with silver nanoparticles for controlled release of anticancer drug

**DOI:** 10.1038/s41598-025-22729-1

**Published:** 2025-10-14

**Authors:** Shaimaa M. Nasef, Ehab E. Khozemy, Ghada A. Mahmoud

**Affiliations:** Radiation Research of Polymer Chemistry Department, National Center for Radiation Research and Technology, Egyptian Atomic Energy Authority, Cairo, Egypt

Retraction of: *Scientific Reports* 10.1038/s41598-023-34870-w, published online 15 May 2023

The Editors have retracted this Article.

After publication of this Article, concerns were raised over inconsistencies between data reported in some tables and figures and the text, including but not limited to the following:

- Table 1 includes a range of pH (3, 4, 5, 5.2, 6, 7, 7.4, and 8) but the Article only reports two pH (1.5 & 7.4);
- the description of the silver nitrate solution relating to the data in Table 2 does not correspond to its description in Table 2;
- the values in Fig. 11B do not match their description in the text.

The Authors provided partial raw data on request, where further issues were found. The Editors have therefore lost confidence in the integrity of the results reported in this Article. All Authors disagree with this retraction.

